# Open Reading Frame 3 of Genotype 1 Hepatitis E Virus Inhibits Nuclear Factor-κappa B Signaling Induced by Tumor Necrosis Factor-α in Human A549 Lung Epithelial Cells

**DOI:** 10.1371/journal.pone.0100787

**Published:** 2014-06-24

**Authors:** Jian Xu, Fan Wu, Deying Tian, Jingjing Wang, Zizheng Zheng, Ningshao Xia

**Affiliations:** 1 Department of Infectious Disease, Tongji Hospital, Tongji Medical College, Huazhong University of Science and Technology, Wuhan, China; 2 Department of Anesthesiology, Tongji Hospital, Tongji Medical College, Huazhong University of Science and Technology, Wuhan, China; 3 National Institute of Diagnostics and Vaccine Development in Infectious Disease, School of Public Health, Xiamen University, Xiamen, China; Virginia Polytechnic Institute and State University, United States of America

## Abstract

Hepatitis E virus (HEV) is one of the primary causative agents of acute hepatitis, and represents a major cause of severe public health problems in developing countries. The pathogenesis of HEV is not well characterized, however, primarily due to the lack of well-defined cell and animal models. Here, we investigated the effects of genotype 1 HEV open reading frame 3 (ORF3) on TNF-α-induced nucleus factor-κappa B (NF-κB) signaling. Human lung epithelial cells (A549) were transiently transfected with ORF3 containing plasmids. These cells were then stimulated with TNF-α and the nucleus translocation of the p65 NF-κB subunit was assessed using western blot and laser confocal microscopy. DNA-binding activity of p65 was also examined using electrophoretic mobility shift assay (EMSA), and the suppression of NF-κB target genes were detected using real-time RT-PCR and ELISA. These results enabled us to identify the decreased phosphorylation levels of IKBα. We focused on the gene of negative regulation of NF-κB, represented by TNF-α-induced protein 3 (TNFAIP3, also known as A20). Reducing the levels of A20 with siRNAs significantly enhances luciferase activation of NF-κB. Furthermore, HEV ORF3 regulated A20 primarily via activating transcription factor 6 (ATF6), involved in unfolded protein response (UPR), resulting in the degradation or inactivation of the receptor interacting protein 1 (RIP1), a major upstream activator of IKB kinase compounds (IKKs). Consequently, the phosphorylation of IKBα and the nucleus translocation of p65 are blocked, which contributes to diminished NF-κB DNA-binding activation and NF-κB-dependent gene expression. The findings suggest that genotype 1 HEV, through ORF3, may transiently activate NF-κB through UPR in early stage, and subsequently inhibit TNF-α-induced NF-κB signaling in late phase so as to create a favorable virus replication environment.

## Introduction

Hepatitis E virus (HEV) infection has become a substantial public health problem all over the world [1]. Transmission of this disease occurs not only through the fecal-oral route [2], but also through blood transfusion [3], person-to-person contact [4], vertical transmission from infected mothers to infants [5], through organ transplantation [6], and zoonosis [7]. Hepatitis E (HE) is associated with high mortality (26.9%) among pregnant women [8], and can result in chronic liver disease in both immunocompromised [9] and immunocompetent individuals [10]. Currently, HEV is divided into 4 genotypes [11], with HEV genotype 1 infection associated with relatively high incidence of viremia and a more severe course than other genotype infections [12]. HEV has three open reading frames (ORFs). ORF1 encodes a nonstructural protein, ORF2 encodes the capsid protein, and ORF3 protein contains two hydrophobic domains (D1, D2) at the N-terminus and two proline-rich domains (P1, P2) at the C-terminus [13]. The detailed role of ORF3 remains obscure. The primary purpose of this study was to characterize molecular events regulated by genotype1 HEV ORF3 at the cell level.

The endoplasmic reticulum (ER) is involved in protein modification, Glucose-regulated protein 78 (GRP78) is defined as an ER stress (ERS) indicator [14]. HEV localizes to the ER [15]. However, the role of HEV ORF3 in the initiation of ERS and subsequent effects remain to be explored. Nuclear factor-κappa B (NF-κB) family members include Rel A (p65), Rel B, c-Rel, p105/50, and p100/p52. In the inactive state, NF-κB remains in the cytoplasm associated with inhibitory proteins called inhibitors of NF-κB (IKB), a family containing IKBα, IKBβ, IKBγ, IKBε, Bcl-3, p100, and p105 [16]. The tumor necrosis factor alpha (TNF-α) has been found to activate NF-κB, and upon exposure to nuclear localization signals, p65 is translocated into the nucleus to bind with a specific DNA sequence and initiate gene transcription [17]. During this event, IKBα is activated and phosphorylated by IKBα kinases (IKKs) consisting of IKKα, IKKβ and IKKγ (also named NEMO) [16]. IKKβ plays a critical role in TNF-α-induced NF-κB activation [18], and RIP1, a major upstream activator of IKKs, is required for the activation of NF-κB pathway [19]. A20, also known as TNF-α-induced protein 3 (TNFAIP3), can terminate NF-κB signaling [20]. NF-κB signaling mediates almost all infectious disease [21], but limited data are available regarding the involvement of HEV ORF3 in the NF-κB pathway because of the lack of an established *in vitro* model. Human A549 lung epithelial cells (A549) have been reported to successfully propagate HEV [22], and therefore represent an appropriate cell line to investigate HEV signal transactivation [23].

In the present study, we investigated the inhibition of TNF-α-induced NF-κB signaling by HEV ORF3 via the unfolded protein response (UPR) in A549 cells. Our research expanded the knowledge regarding HEV ORF3 biology *in vitro*, and elucidated mechanisms of ORF3-mediated modulation of cellular processes involved in HEV infection.

## Materials and Methods

### Plasmids and Biological Reagents

The HEV ORF3 fragment was PCR-amplified from the Sar-55 gene, which was a gift from the National Institute of Diagnostics and Vaccine Development in infectious diseases (NIDVD, China). It was cloned into the pEGFP-N1 vector (GFP, GenBank accession #55762) by digesting with *Bam*HI and *Hind*III to construct the ORF3-EGFP plasmid (pORF3). Mutant ORF3 clones were constructed using the overlapping-PCR technique. Briefly, mutant D1 domain (ΔD1) was constructed by deleting 15–31 amino acids (aa), and the mutant D2 (ΔD2) was constructed by removing 37–62 aa from pORF3. Mutants P1 (ΔP1) and P2 (ΔP2) were constructed by changing proline into alanine from pORF3, respectively. All the clones were confirmed by restriction digestion and DNA sequencing. Primers are shown in Table 1. The infectious cDNA clone of the Sar-55 strain was kind gifted from Dr. SU. Emerson (NIH, USA) [24].

**Table 1:** Primers and probes.

Name	Direction	Primer
Sar55	Forward	CCCAAGCTTACCATGAATAACATGTCTTTTGC
	Reverse	CGC GGATCCGCGCGGCGCGGCCCCA
ΔD1	Forward	ATGGGTTCGCGACCACCGCGCCACCGCCCG
	Reverse	CGGGCGGTGGCGCGGTGGTCGCGAACCCAT
ΔD2	Forward	CCGCGCCACCGCCCGAGCCCTTCGCAATCC
	Reverse	GGATTGCGAAGGGCTCGGGCGGTGGCGCGG
ΔP1	Forward	TCCAACCAACCGCATCGGCAGCAATGTCAGC ACTGCGGGCAGGGCTGGACCTCGTGTT
	Reverse	AACACGAGGTCCAGCCCTGCCCGCAGTGCTGA CATTGCTGCCGATGCGGTTGGTTGGA
ΔP2	Forward	TGCCCCCAGCTGTGCTAGGTCTACGACGTGTG CCAGTGCTGCGGCGCTTGCCCTGGTCA
	Reverse	CGC GGATCCGC GCGGCG TGCCCCCAGCTGTGCTAG
Consenus NF-κB probe	Forward	AGTTGAGGGGACTTTCCCAGGC
	Reverse	TCAACTCCCCTGAAAGGGTCCG
Mutant NF-κB probe	Forward	AGTTGAGGCGACTTTCCCAGGC
	Reverse	TCAACTCCGCTGAAAGGGTCCG
A20	Forward	GGGTGGAATTTACTTGCC
	Reverse	AGGGTCACCAAGGGTACA
RIP1	Forward	AAATGCAGTTGTGAAGAG
	Reverse	TTGACCGGCTTGAAGGTA
IL1β	Forward	ACGAATCTCCGACCACCACTA
	Reverse	GCACATAAGCCTCGTTATCCC
COX2	Forward	CAAGTCCCTGAGCATCTACGG
	Reverse	TGATAGCCACTCAAGTGTTGCA
ICAM1	Forward	AAGGATGGCACTTTCCCACT
	Reverse	GTGATGATGACAATCTCATACCG
β-actin	Forward	ACAGAGCCTCGCCTTTGC
	Reverse	ATCATCCATGGTGAGCTG

We obtained p65, IκBα, phosphorylated IκBα (Ser32/36), IKKβ, A20, RIP1, and GRP78 primary antibodies from R&D Systems (Minneapolis, MN, USA). 4-(2-Aminoethyl) benzenesulfonyl fluoride (AEBSF) and tunicamycin were purchased from Sigma (Sigma, Aldrich, Japan). 1, 2-dimyristyloxypropyl-3-dimethy1-hydroxy ethy1 ammonium bromide and cholesterol (DMRIE-C) were obtained from Invitrogen (Invitrogen Carlsbad, CA). Recombinant purified human TNF-α (Peprotech, New Jersey, USA), HEV ORF3 primary antibody (Beijing Protein Innovation Co. Ltd, China), ATF6 primary antibody (Imgenex, San Diego, CA, USA) and horseradish peroxidase (HRP)-tagged secondary antibody (Santa Cruz, CA) were obtained commercially.

### Cell Culture, Transfection, and Treatment

A549 and Huh7 cell lines were purchased from American Type Culture Collection (Manassas, VA). A549 cells were maintained in RPMI 1640 media containing 10% fetal bovine serum (FBS), penicillin (100 µg/ml) and streptomycin (100 µg/ml) and incubated at 5% CO_2_ and 37°C. For Huh7 cells, Dulbecco’s modified Eagle’s medium culture media were used. Cells were grown to 60%–80% density in 6-wells plate, and transfected using DMRIE-C reagent according to the protocol. The ratio of plasmid to DMRIE-C was 2 µg: 8 µl. At 48 h post-transfection, cells were exposed to 50 ng/ml of TNF-α (diluted in the RPMI 1640 medium with 10% FBS) for 6 h, and RNA or protein was extracted as needed.

### Western Blot

Cells were lysed with 4, 4′-diaminodiphenylmethane (DDM), and cell lysates containing 1 M HEPES, l00 Mm EDTA, 250 mM MgCL_2_, 5 M NaCl and DDM powder, were obtained. The supernatants were collected and quantified by the Bradford protein assay (Bio-RAD Laboratories, Hercules, CA, USA). Nuclear and cytoplasmic protein extracts (BOSTER, Wuhan, China) were obtained, according to the protocol [25]. Equal amounts of proteins (20 µg) were resolved by 12% sodium dodecy1 sulfate-polyacrylamide gels electrophoresis (SDS-PAGE) and transferred onto PVDF membranes (EMD Millipore). The membranes were blocked with 5% skimmed milk (diluted in PBS) at room temperature for 1 h, and incubated with appropriate primary antibodies [diluted l: 1,000 with 1×Tris-NaOH (TN)]. The membranes were cleaned 3 times with TNT (TN containing 0.1% Tween-2000) followed by incubation with HRP-linked secondary antibody (diluted l∶5,000 in 1×TN) for 1 h at room temperature and washed 3 times as described above. The GAPDH or Lamin B was used as a loading control. All protein bands were analyzed using chemiDoc™ MP Imaging System (Bio-RAD Laboratories, Hercules, CA, USA).

### Immunofluorescence Laser Confocal Microscopy Assay

Coverslips were seeded in 24 wells plate before cells were seeded. Cells were transfected and treated as above. At indicated times, cells were washed 3 times with phosphate buffered saline (PBS), 4% pre-cold paraformaldehyde was used to fix the cells in dark room at room temperature, rinsed 3 times with PBS and mixed with 0.3% Triton (diluted in PBS) at room temperature for 10 min, washed 3 times again. Cells were incubated with 3% normal goat serum for 1 h. Coverslips were incubated with a p65 monoclonal antibody (l∶250 dilution with PBS) for 1 h and incubated in the dark with fluorescein isothiocyanate (FITC) tagged secondary antibody for 1 h. Coverslips were incubated with 4′,6′-diamidino-2-phenylindole (DAPI, l∶2000 dilution with PBS) for 5 min and washed 3 times. Dropped coverslips on a tablet and sealed around with nail oil, then observed with laser confocal microscope (Beckman, USA).

### Electrophoretic Mobility Shift Assay (EMSA)

Biotin-labeled NF-κB and mutant NF-κB probes were purchased from Beyotime (Nantong, China). Probes were shown in Table 1. 10 µg of nuclear proteins of each specimen were incubated with 50 fmol of the biotin-labeled probe for 30 min at room temperature in the presence of 1 µg of poly (dI: dC). Protein-DNA complexes were separated from the free DNA probe by electrophoresis via a 6.5% native polyacrylamide gel. The gel was run in the 0.5×Tris Borate Ethylene diamine tetraacetic acid (TBE) at room temperature with 30 mA for 2.5 h. The separated proteins were transferred to PVDF membranes (EMD Millipore) at 380 mA for 30 min, cross-linked under the ultraviolet lamp for 10 min, and subjected to gentle shaking in sealing liquids containing streptavidin-HRP conjugate for 15 min. The membrane was washed and balanced, followed by addition of 5 ml BeyoECL plus Reagent A and 5 ml BeyoECL plus Reagent B. Proteins were finally analyzed with chemiDoc™ MP Imaging System (Bio-RAD). Competition tests used a 50-fold excess of the unlabeled oligoduplex.

### Real-time Reverse Transcriptase-Polymerase Chain Reaction (RT-PCR)

Total RNA was extracted using the Trizol reagent (Invitrogen, Carlsbad, CA, USA) and reverse transcribed to cDNA using reverse transcription system (Promega, Madison, WI, USA). 1 µl of cDNA was combined with primers and SYBR Green PCR Master Mix (CT biosciences, China) to yield a final volume of 30 µl. The PCR conditions included 40 cycles of 94°C for 30 s, 58°C for 30 s, and 72°C for 5 min on a StepOnePlus™ Real-Time PCR System (Applied Biosystems, CA, USA). Threshold cycle (*CT*) values were determined by RT-PCR and normalized by the housekeeping gene â-actin. The relative level of indicated genes was calculated using the 2^−ΔΔCt^ method. Primers are shown in Table 1.

### Enzyme Linked Immunosorbent Assay (ELISA)

At the indicated time, culture supernatants were harvested. The secretion of IL-1β, COX2 and ICAM-1 was measured with ELISA (Uscnlife science, Wuhan, China). The proteins were quantified, based on the standard curve obtained with purified protein in the ELISA kit.

In conclusion, these findings imply that HEV, through ORF3, may suppress NF-κB signaling pathway and implicate a mechanism by which HEV may escape from host’s immune clearance, leading to a favorable condition for its own replication in host cells. Although there is no research *in vivo* suggesting that the main observation is physiologically relevant, the data *in vitro* expand a novel biologic role of HEV ORF3. Further efforts will be conducted to identify whether NF-κB signaling were affected *in vivo.*

### RNAi Assay

A549 cells were transfected with a scrambled control or with commercial short interfering RNA (siRNA) against A20 (Cat. #4309771, Ambion, USA), according to DMRIE-C reagent protocol.

### Luciferase Assay

Luciferase activity of NF-κB was evaluated by the luciferase assay system (Promega, Madison, Wisconsin). A549 cells were co-transfected with pNF-κB-Luc and the pSV-β-galactosidase plasmids with or without pORF3 using DMRIE-C reagent. At 48 h post-transfection, cells were exposed to TNF-α for 6 h. Preparation of total protein was described above. The 20-µl mixture containing 8 µg of each sample extract and Reporter Lysis Buffer was used to detect luciferase activity. One-hundred microliters of Luciferase Assay Reagent was added to each diluted extract and measured on a Beckman coulter (Beckman, Germany). The β-galactosidase luciferase served as an internal control.

### Statistical Analysis

The results were expressed as mean ± standard deviation (SD). Statistical analysis was performed by one-way ANOVA for overall significance followed by the Tamhane’s T2 (M) test. P<0.05 was considered statistically significant. All analyses were carried out with the SPSS 20.0.

## Results

### Activation of NF-κB

**Figure 1:**
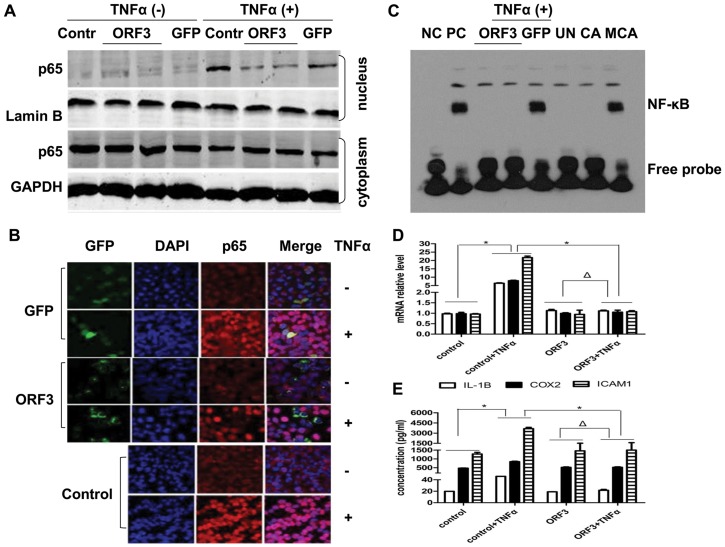
Activation of NF-κB. (A) A549 cells were transfected with either pORF3-EGFP (ORF3) or pEGFP-N1 (GFP) for 48 h, stimulated with TNF-α (50 ng/ml) for 6 h, and then subjected to western blotting, using the untreated cells as a control (Contr). (B) Immunofluorescence laser confocal microscopy for the detection of p65. A549 cells were transfected with either pORF3-EGFP (ORF3) or pEGFP-N1 (GFP) for 48 h and then stimulated with TNF-α (50 ng/ml) for 6 h, with another untreated group regarded as the control. The distribution of p65 was examined using p65-specific monoclonal antibody (red). The nucleus was counterstained with DAPI and observed under a confocal laser scanning microscope (magnification: 100×). (C) TNF-α induced NF-κB DNA-binding activity in pORF3-expressing cells. After 6 h of TNF-α treatment, the nuclear extracts were used to determine the NF-κB activity by EMSA. NC, negative control; PC, positive control; ORF3, transfected with pORF3; GFP, transfected with empty pEGFP-N1; UN, untreated cells; CA, competition assay; MCA, mutant competitive assay. (D) Effects of pORF3 on TNF-α-induced NF-κB target genes by real-time RT-PCR. The relative expression of IL-1β, COX-2, and ICAM-1 was calculated following normalization to β-actin, and fold changes relative to the expression levels in untreated cells (control) are presented. (E) The levels of IL-1β, COX-2, and ICAM-1 secreted in the supernatant of the culture medium were detected by ELISA. The protein values used for the calculation were obtained from a corresponding standard curve. The data are presented as mean ± SD from three independent experiments (each performed in triplicate). *P<0.05, ΔP>0.05.

At 48 h post transfection, cells showed 10–15% green fluorescent by fluorescence microscopy indicated that ORF3 was successful expressed in A549 cells (Figure S1). To observe the effect of pORF3 on TNF-α-induced NF-κB activation, we determined the expression of cytoplasmic and nuclear p65 using western blot analysis. We found that p65 remained in the cytoplasm when not stimulated by TNF-α, but it translocated into the nucleus in the control and GFP groups following TNF-α stimulation. However, it was only weakly translocated into the nucleus in pORF3-expressing cells (Figure 1A). To examine whether ORF3 has the same potential in liver cells, Huh7 cells were transfected with pORF3 or GFP. As is shown in Figure S2 A, ORF3 protein was expressed and localized to plasmatic in Huh7 cells. The suppression of p65 nuclear translocation by ORF3 was observed in Huh7 cells besides in A549 cells (Figure S2 B). The same phenomenon was verified with immunofluorescence laser confocal microscopy assay (Figure 1B). We next determined whether the DNA binding activity of NF-κB was reduced following pORF3 transfection. Nuclear extracts were prepared for examination of NF-κB activity by EMSA. Unlike the negative control, the GFP group and the positive control, showed a protein-DNA complex under TNF-α stimuli. Cells expressing pORF3 protein lacked the complex, irrespective of TNF-α stimulation. This complex was abolished by excess cold probe, but remained unchanged with a mutant probe (Figure 1C). To further evaluate the effect of pORF3 on TNF-α-induced NF-κB dependent gene expression, three NF-κB target genes, IL-1β, COX2 and ICAM-1 were analyzed by real-time RT-PCR. In the absence of pORF3, TNF-α stimulation enhanced mRNA expression of all three genes compared with the absence of TNF-α (P<0.05). No difference was seen between with TNF-α stimulation and without in pORF3-pretreated cells (P>0.05). The difference after TNF-α stimulation of pORF3-pretreated cells was statistically significant compared with the absence of pORF3-pretreated group (P<0.05) (Figure 1D). Similar results for levels of protein expressed from these three genes were also confirmed by ELISA (Figure 1E).

### IKBα Phosphorylation

**Figure 2:**
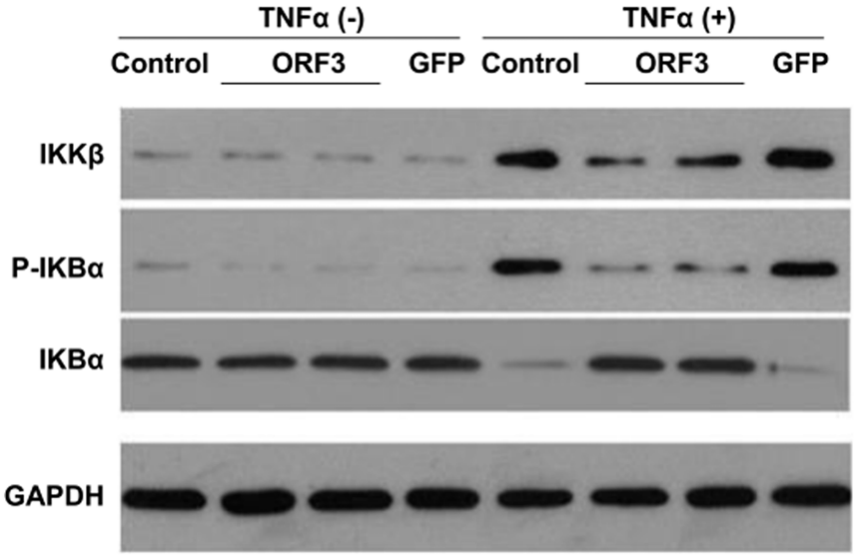
IKBα phosphorylation. A549 cells were transfected with 2 µg of either pORF3-EGFP (ORF3) or pEGFP-N1 empty vector (GFP), and untreated cells were used as the control. After 48 h, the cells were exposed to 50 ng/ml TNF-α for 6 h. The total proteins extracted from these cells were subjected to western blotting with anti-IKBα, anti-phosphorylated IKBα (P-IKBα), and anti-IKKβ antibodies. GAPDH was used as a loading control.

Following TNF-α stimulation, lower levels of phosphorylated IKBα were observed in pORF3-expressing cells than those in pORF3 unexposed cells (Figure 2). Suppressed activation of IKKβ was also seen in the pORF3-expressing cells compared to controls.

### ORF3 Curbs TNF-α-induced NF-κB Signal through A20

**Figure 3:**
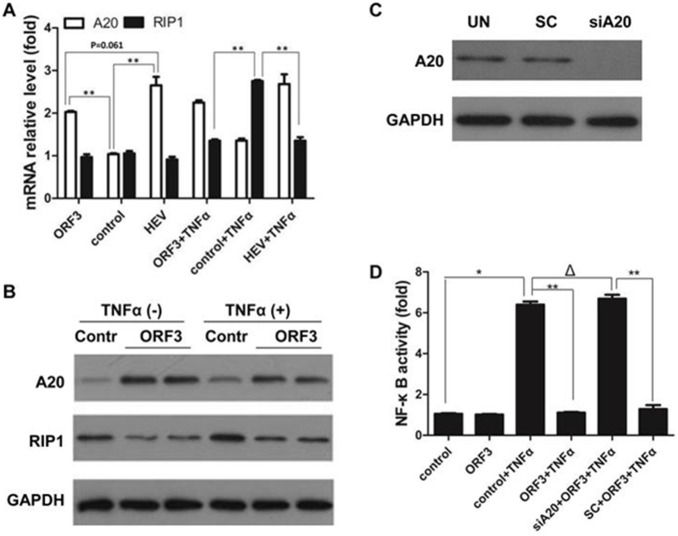
ORF3 abrogated TNF-α-induced NF-κB signal via A20. (A) A549 cells were transfected with pORF3-EGFP (ORF3) or an infectious cDNA clone of HEV (HEV), after 48 h, these cells were treated with 50 ng/ml TNF-α for 6 h. The relative mRNA expression levels of A20 and RIP1 were detected by real-time RT-PCR. Untreated cells served as a control. Relative values against β-actin were calculated, and fold changes relative to the control are provided. The results are representative of three independent experiments (each performed in triplicate). **P<0.01. (B) Western blot of A20 and RIP1. (C) Scrambled control (SC) or siRNA to A20 (siA20) were transfected into A549 cells, and cells were harvested after 24 h. Untreated cells were used as an empty vector control (UN). Total protein extracted from the harvested cells was used to evaluate A20 expression by western blot, using GAPDH as a loading control. (D) A549 cells were transiently transfected with pNF-κB-Luc, pretreated with or without pORF3, exposed to TNF-α for 6 h, and subjected to luciferase assay. The other two groups were exposed to siRNA against A20 (siA20) or scrambled siRNA (SC) for 24 h, respectively, before transfection with pNF-κB-Luc. The cells that were not transfected with pORF3 served as a control. Luciferase activity was normalized to β-galactosidase, and fold changes against control are provided. The results shown are representative of three independent experiments (each performed in triplicate). **P<0.01, *P<0.05, Δ P>0.05.

Real-time RT-PCR was performed to detect levels of the A20. As shown in Figure 3A, A20 exhibited a basal expression in A549 cells, but was up-regulated in either pORF3 or HEV pretreated cells compared with controls (P<0.01). The levels of A20 have no difference between pORF3-pretreated and HEV expressed cells (P = 0.061). Under TNF-α stimulation, RIP1 showed a significant increase in the group neither pORF3 nor HEV pretreated (P<0.01). Western blot analysis was carried out to confirm the correlation between A20 and RIP1 in pORF3-pretreated cells (Figure 3B). We also examined the role of A20 with commercial siRNA. We first tested the ability of siRNA to interfere the expression of A20 (Figure 3C), and a subsequent luciferase assay indicated that pORF3 failed to activate the NF-κB signal, even with TNF-α stimulation. However, siRNA against A20 abrogated the suppressive effects of pORF3 on TNF-α-induced NF-κB activation. The difference was statistically significant compared to control scrambled siRNA (P<0.01) (Figure 3D).

### UPR Participated in the Up-regulation of A20

**Figure 4:**
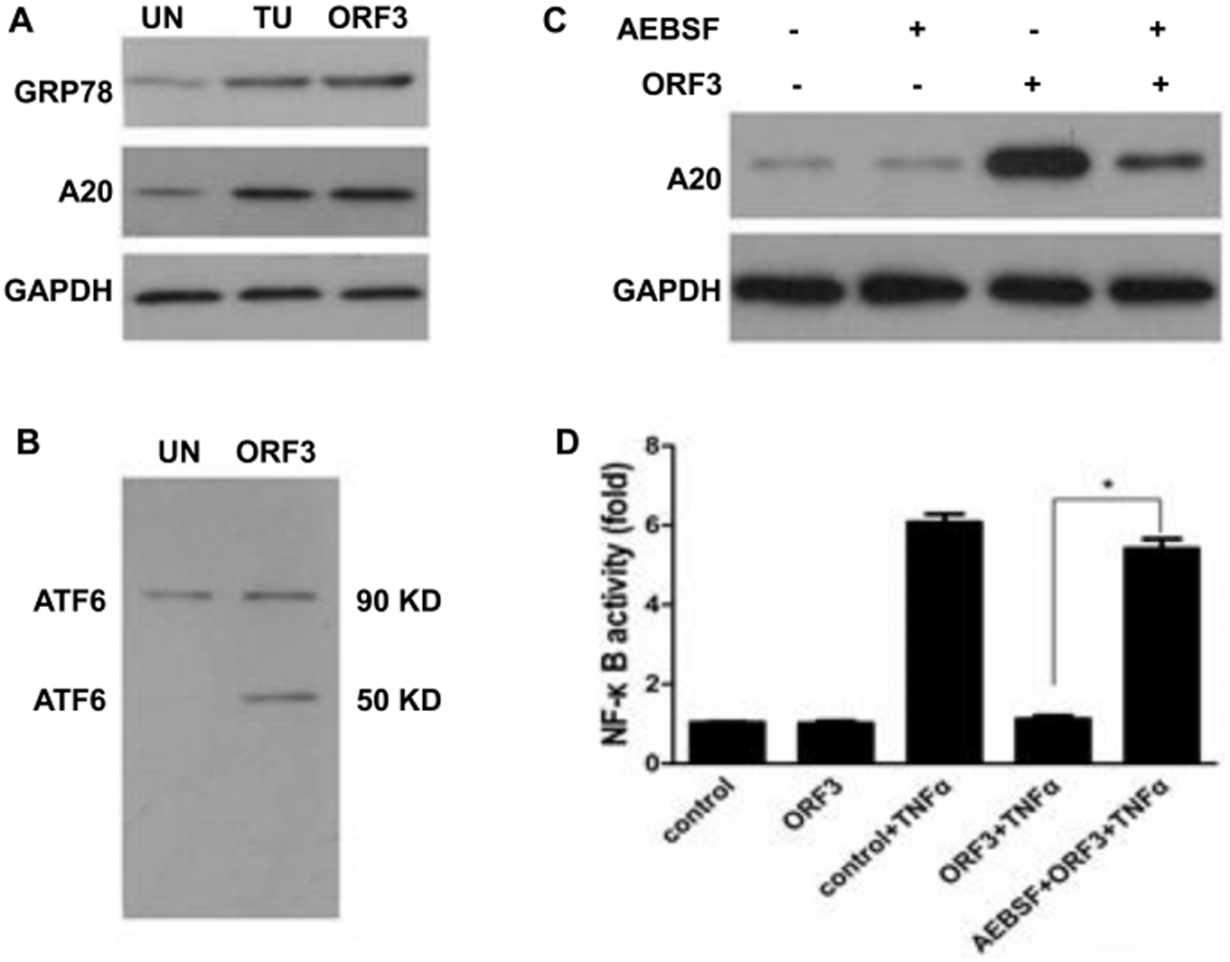
UPR participated in the up-regulation of A20. (A) Cells were treated with tunicamycin (TU; 2 µg/ml), pORF3, or were untreated (UN) for 48 h, and subjected to western blotting. (B) Spliced ATF6 was detected in A549 cells with or without pORF3 pretreated by western blotting. (C) A549 cells were pretreated with (+) or without (−) 250 µM AEBSF for 1 h and then transfected with pORF3 or not. Protein extracts were processed for western blotting by using A20 antibody. (D) Cells were co-transfected with pNF-κB-Luc and pORF3 for 48 h, and the other group that was transfected only with pNF-κB-Luc served as a control. Both groups were exposed to TNF-α for 6 h and subjected to a luciferase assay. Another group was exposed to AEBSF (250 µM) before co-transfection with pNF-κB-Luc and pORF3. Luciferase activity was normalized to β-galactosidase, and fold changes against the control are presented. The results are representative of three independent experiments (each performed in triplicate). *P<0.05.

We further analyzed the UPR in pORF3-expressing cells. A20 and GRP78 showed basal expression in A549 cells, which was further enhanced by treatment with pORF3. The effect of pORF3 in these cells reflected treatment with tunicamycin, an activator of UPR [26] (Figure 4A), and we found that ATF6 was spliced into ATF6 90 KD and ATF6 50 KD in pORF3-pretreated cells (Figure 4B). We speculated that ATF6 is involved in the induction of A20 by pORF3. To test this hypothesis, A20 levels were evaluated in A549 cells with or without pORF3 or AEBSF, an inhibitor of ATF6. Western blot analysis revealed that the suppression of ATF6 by AEBSF abrogated the enhancement of A20 induced by pORF3 (Figure 4C). AEBSF pretreatment also abolished the inhibition of TNF-a-induced NF-κB activation by pORF3 (Figure 4D).

### P2 Domain Played the Inhibitory Effect

**Figure 5:**
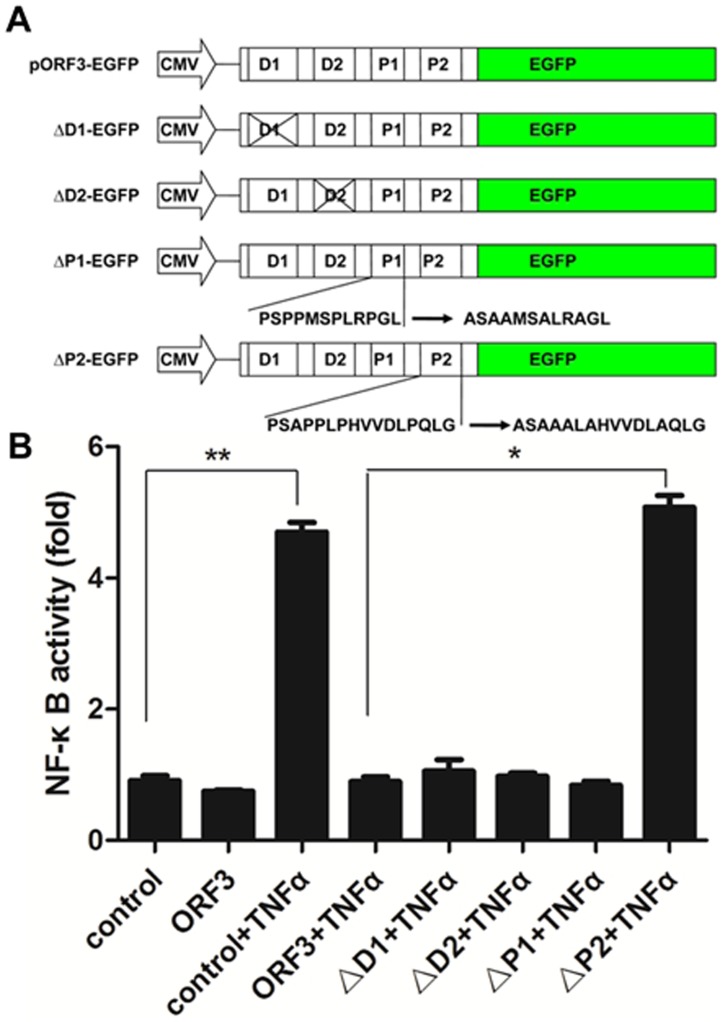
P2 domain played the inhibitory effect. (A) Schematic illustration of expression vectors used in this study. The pEGFP-N1 vector (Clontech) carries the EGFP gene of a cytomegalovirus (CMV) promoter and multiple cloning sites. The plasmid of pORF3-EGFP containing the 123 amino-acid (aa) ORF3 lacking its stop codon was cloned upstream of the EGFP gene. This plasmid produces a 123-aa ORF3-EGFP fusion protein. Mutant plasmids of D1-EGFP (ΔD1) and D2-EGFP (ΔD2) were constructed by deleting (x) D1 and D2 domain, respectively. Mutant plasmids of P1-EGFP (ΔP1) and P2-EGFP (ΔP2) were constructed by changing proline (P) into alanine (A), respectively. (B) A549 cells were co-transfected with NF-κB reporter plasmid and pORF3 or one of the mutant plasmids for 48 h, exposed to TNF-α (50 ng/ml) for 6 h, and subjected to a luciferase assay. Luciferase activity was normalized to β-galactosidase, and fold change values were compared to the untreated cells (control). The results shown are representative of three independent experiments (each performed in triplicate). *P<0.05, **P<0.01.

NF-κB activity was analyzed by co-transfecting A549 cells with NF-κB reporter plasmid and either pORF3 or its mutant domains. As shown in Figure 5, stimulation with TNF-α led to increased luciferase expression in mutant P2 protein (ΔP2) compared with to pORF3-expressing cells (P<0.05), suggesting that the P2 domain is critical in the regulation of TNF-α-induced NF-κB activity.

## Discussion

HEV, is classified as a member of *Hepevirus* belonging to the *Hepeviridae* family [27], represents the known cause of HE [28]. Increased incidences of indigenous HE cases have been reported in developing and developed countries [29]. Although a recombinant vaccine preventing from HEV infection is available in China [30], the pathogenesis of HE remains unclear.

It is reported that liver injury in HE patients is immune mediated [31], and immune clearance is the major manner of host cells to resist microbial invasion [32]. However, the exact mechanism of HEV in escaping the host immune surveillance is not yet known. Many viruses, such as poxviruses [33], Hepatitis C virus [34], and human immunodeficiency virus [35], are thought to regulate the activation of NF-κB during infection to balance the replication environment and virus survival. Previous reports have shown that HEV ORF3 is involved in the regulation of multiple signaling pathways [36], such as those involving the epidermal growth factor receptor (EGFR) [37], extracellular regulated kinase (ERK) [38] and interferon [23]. However, no conclusive evidence supporting the role of HEV ORF3 in NF-κB signaling was previously available due to the absence of an efficient HEV cell model *in vitro.* Propagation of HEV in A549 cells was reported recently [23]. In the present study, we constructed an ORF3-GFP fusion protein expressed successfully in A549 cells and Huh7 cells.

Nuclear translocation of p65 indicates NF-κB activation, and we found that TNF-α-induced p65 nuclear translocation was effectively inhibited by pORF3 not only in A549 cells but also in liver cells including Huh7 cells. The active form of p65 binds specifically to its DNA consensus site and forms a DNA-protein complex, and EMSA data from our investigation demonstrated that the DNA-binding activity of NF-κB was blocked in pORF3-expressing cells. We propose that the TNF-α-induced NF-κB activation was inhibited. Constitutive activation of NF-κB correlates with the expression of immune response molecules [39]. We explored the mRNA and protein expression levels of the NF-κB mediated genes, including IL-1β, COX2 and ICAM-1. These effects revealed that levels of IL-1β, COX2 and ICAM-1 were lowered by pORF3 due to suppressed NF-κB activation, which suggested that pORF3 attenuated TNF-α-induced NF-κB activation. We also found that the P2 domain was critical for the inhibition of NF-κB by pORF3, although the respective mechanism for this inhibition remains unclear.

Nuclear translocation of p65 depends on the phosphorylation of IκBα resulting from the activation of IKKβ [40], and our research shows decreased IKKβ activation and a concomitant reduction in TNF-α-induced phosphorylation in pORF3-expressing cells. These results suggest that pORF3 protein plays an inhibitory role in the IKKβ–IκBα-NF-κB cascade. To elucidate mechanisms underlying this regulation, we also investigated the role of A20, a negative regulator of NF-κB. Our experiments showed enhanced A20 levels in both pORF3-expressing cells and HEV pretreated cells. These results are consistent with a previous report [41]. It is logical to hypothesize that ORF3 terminates the TNF-α-induced NF-κB signaling via A20. We also observed a simultaneous disruption of TNF-α-induced RIP1 activation following pORF3 or HEV treatment. Under TNF-α stimulation,RIPl normally activates IKKγ followed by NF-κB activation [42], and A20 acts as a dual-function ubiquitin-editing enzyme to modify RIP1 and negatively modulate its function [43]. The amino terminal domain of A20 removes K63-linked ubiquitin chains from RIP1 and the C-terminal domain acts as a ubiquitin ligase, adding K48-linked polyubiquitin chains on RIP1 to catalyze proteasomal RIP1 degradation [44]. In this study, RNAi and luciferase assays demonstrated that pORF3-pretreated cells merely exhibited basal luciferase activation even under TNF-α stimulation. However, enhanced luciferase activation was observed in A20-silenced cells under the same conditions. Our results suggested that RIP1 may be degraded by redundant A20 induced by pORF3.

An overload of protein folds leading to the activation of NF-κB [45], excessive unfolded protein in ER trigger an unfolded protein response (UPR) [46]. Three ER protein sensors including the inositol requiring enzyme 1 (IRE1), double-stranded RNA-activated protein kinase (PKR)-like ER kinase (PERK) and activating transcription factor 6 (ATF6) have been identified to date [47]. ERS is regarded as an inducer of NF-κB signaling [48]. Enhanced GRP78 level in pORF3-expressing cells suggested that pORF3 may cause UPR. In response to UPR, ATF6 translocates to the Golgi apparatus, where it is spliced by site 1/2 protease (S1/2P) into ATF6 p90 and ATF6 p50. The translocation of ATF6 p50 into the nucleus is followed by initiation of specific gene expression [49]. ATF6 was spliced in pORF3-expressing cells also suggested that pORF3 initiated UPR. The ATF6 contains a b-Zip DNA transcription domain at its N-terminus [50], and therefore plays a critical role in promoter activation [51]. Our research found that A20 was associated with the activation of ATF6 in pORF3-expressing cells. To confirm the role of ATF6, we used AEBSF, an inhibitor of ATF6 [52]. The results indicated that the successful inhibition of ATF6 by AEBSF decreased the A20 induction triggered by pORF3. This phenomenon supported the idea that the suppression of TNF-α-induced NF-κB signal by pORF3 was dependent on the transacting potential of ATF6.

Currently, it is known that ERS contributes to activation of NF-κB [48], but is unclear whether and how this negatively regulates this pathway. Another report demonstrated that ERS-induced activation of NF-κB was increased by short-term exposure to ERS inducers, but was down-regulated by long-term exposure to ERS inducers [53]. These data indicate that pORF3 initiated transient activation of NF-κB in the early stage, and subsequently, A20, as a downstream protein of NF-κB pathway, was enhanced and exerted a negative modulation. These results pose a possibility that transient ERS induced by pORF3 may activate NF-κB signals through ATF6 pathway and consequently provide negative feedback on NF-κB through enhanced A20 in the late phase.

**Figure 6:**
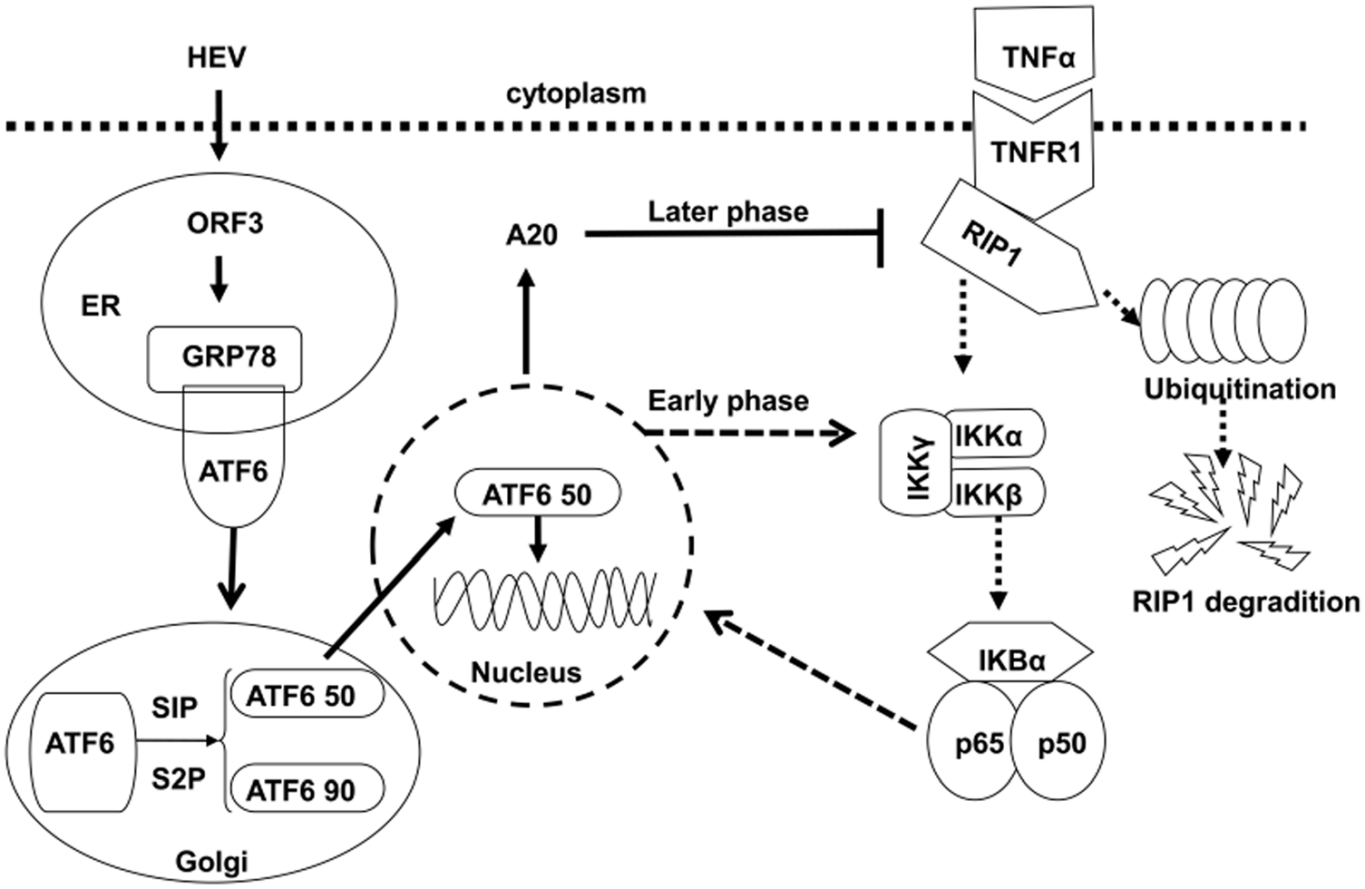
Putative mechanism underlying inhibition of NF-κB by pORF3. The pORF3 activates transiently NF-κB and up-regulates A20 via the ATF6 pathway in the early phase, and causes degradation or inactivation of RIP1 in the late phase, leading to the blockade of the TNF-α-induced NF-κB signaling.

As indicated in Figure 6, our results imply a possibility that pORF3 initiated UPR mediated by ATF6 and contributed to the activity of NF-κB in the early phase. The transient activation of NF-κB signaling might be responsible for the induction of A20. In the later phase, the enhanced A20 was involved in the inhibition of TNF-α-induced NF-κB signal, which suppressed numerous genes involved in inflammatory response.

## Supporting Information

Figure S1 **Transfection efficiency of ORF3 plasmid in A549 cells.** (A) A549 cells were grown to 60–80% density in 6-well plate, and then were transfected. After 48 h from transfection, cells were observed in fluorescence microscopy under normal light. (B) Transfection efficiency of ORF3 plasmid in A549 cells under fluorescence light (magnification: 200×).
(TIF)

Figure S2 **HEV ORF3 suppressed TNF-α induced NF-κB in Huh7 cells.** Expression and localization of pORF3 in Huh7 cells. Huh7 cells were transfected with either pORF3-GFP (ORF3) or pEGFP-N1 (GFP) for 48 h, the ORF3 protein with green fluorescent was observed with laser confocal microscopy (magnification: 1000×). (B) Huh7 cells were transfected with either pORF3-GFP (ORF3) or pEGFP-N1 (GFP) for 48 h, stimulated with TNF-α (50 ng/ml) for 6 h, and then subjected to western blotting, using the untreated cells as a control (UN).
(TIF)
