# Supplementary figures and images for: Open Reading Frame 3 of Genotype 1 Hepatitis E Virus Inhibits Nuclear Factor-κappa B Signaling Induced by Tumor Necrosis Factor-α in Human A549 Lung Epithelial Cells
Source: PLoS One. 2014 Jun 24;9(6):e100787. doi: 10.1371/journal.pone.0100787 (PMC4069097; doi:10.1371/journal.pone.0100787)

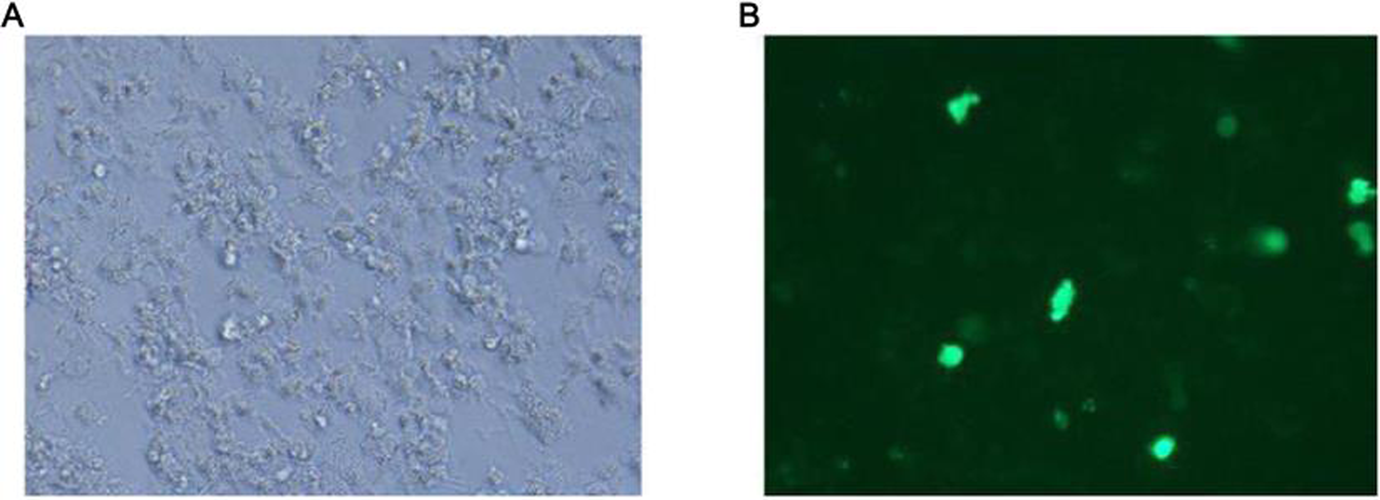

Supplement: Figure S1 — Transfection efficiency of ORF3 plasmid in A549 cells. (A) A549 cells were grown to 60–80% density in 6-well plate, and then were transfected. After 48 h from transfection, cells were observed in fluorescence microscopy under normal light. (B) Transfection efficiency of ORF3 plasmid in A549 cells under fluorescence light (magnification: 200×). (TIF) [file pone.0100787.s001.tif]

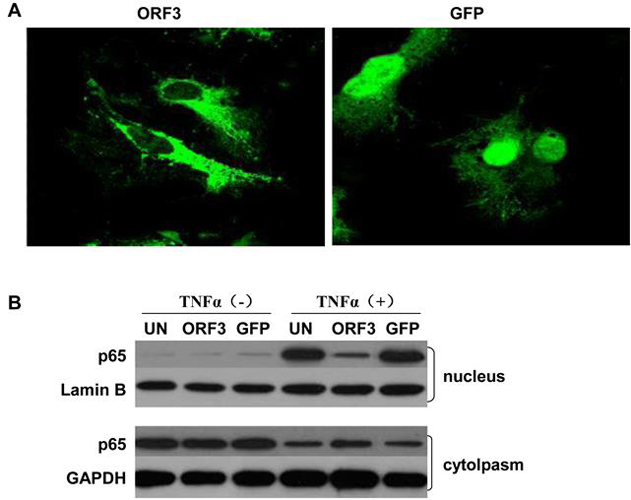

Supplement: Figure S2 — HEV ORF3 suppressed TNF-α induced NF-κB in Huh7 cells. Expression and localization of pORF3 in Huh7 cells. Huh7 cells were transfected with either pORF3-GFP (ORF3) or pEGFP-N1 (GFP) for 48 h, the ORF3 protein with green fluorescent was observed with laser confocal microscopy (magnification: 1000×). (B) Huh7 cells were transfected with either pORF3-GFP (ORF3) or pEGFP-N1 (GFP) for 48 h, stimulated with TNF-α (50 ng/ml) for 6 h, and then subjected to western blotting, using the untreated cells as a control (UN). (TIF) [file pone.0100787.s002.tif]
